# Tracheobronchoplasty for severe tracheobronchomalacia: a case-series of patients with acute and chronic critical comorbidities

**DOI:** 10.1093/icvts/ivae155

**Published:** 2024-09-18

**Authors:** Robert Herron, Ankit Dhamija, Jenna Shumar, Jahnavi Kakuturu, J W Awori Hayanga, Jason Lamb, Alper Toker

**Affiliations:** Department of Cardiovascular and Thoracic Surgery, West Virginia University, Morgantown, WV, USA; Department of Cardiothoracic Surgery, Stony Brook University, Stony Brook, NY, USA; Department of Cardiovascular and Thoracic Surgery, West Virginia University, Morgantown, WV, USA; Department of Cardiovascular and Thoracic Surgery, West Virginia University, Morgantown, WV, USA; Department of Cardiovascular and Thoracic Surgery, West Virginia University, Morgantown, WV, USA; Department of Cardiovascular and Thoracic Surgery, West Virginia University, Morgantown, WV, USA; Department of Cardiovascular and Thoracic Surgery, West Virginia University, Morgantown, WV, USA

**Keywords:** Tracheobronchomalacia, Tracheobronchoplasty, Trachea, Robotic, Airway

## Abstract

**OBJECTIVES:**

There are little data within the literature regarding tracheobronchoplasty in the setting of the acute and chronically ill, morbidly obese or ventilator-dependent patients with tracheobronchomalacia. Short- and long-term outcomes are studied.

**METHODS:**

The series represents 12 tracheobronchomalacia patients with American Society of Anesthesiologists (ASA) physical status scores of 3–5. Candidacy was based on bronchoscopic findings during spontaneous respirations with >90% collapse of the trachea and both mainstem bronchi. We used dynamic computed tomography scan as an adjunct in those not mechanically ventilated. Our operative approach was a complete portal robotic approach for those outpatients (wheelchair dependent) and right thoracotomy for those who were already mechanically ventilated with 100% fraction of inspired oxygen with high pressure. Extracorporeal support was used in 2 patients.

**RESULTS:**

Patients who underwent robotic repair were discharged without complications. Two patients who were critically ill and required extracorporeal support for their surgeries were separated from extracorporeal membrane oxygenation on postoperative day 2. Three patients died at the follow-up. In 1 patient, the prolene mesh migrated into trachea and caused obstruction of the trachea and required removal with endobronchial techniques.

**CONCLUSIONS:**

The repair of tracheobronchomalacia in patients with multiple comorbidities and with severe life-threatening problems in or outside the intensive care unit may have improvement due to the ability to wean from positive pressure ventilation. Surgical technique and the utilization of mesh support in tracheobronchoplasty operations may need to be debated due to duration of the surgery in patients with severe comorbidities.

## Graphical Abstract

**Figure:**
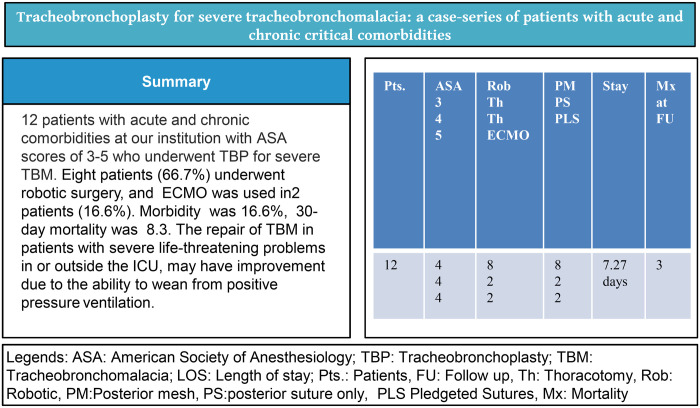

## INTRODUCTION

Tracheobronchomalacia (TBM) in adults is typically managed conservatively with positive airway pressure therapy or stents. However, symptomatic TBM sometimes requires surgical correction if these more conservative modalities fail [1]. Severe TBM is increasingly recognized as a cause for respiratory failure in the intensive care unit (ICU). Physiologically, morbid obesity and the resulting increased intrathoracic pressure, particularly in the supine position, contribute to an exaggeration of normal expiratory airway collapsibility [2]. The surgical correction of TBM involves tracheobronchoplasty (TBP). This operation is most commonly performed in the elective setting in patients who are not acutely ill and/or ventilator dependent. Thus, there is a paucity of date describing the performance of tracheobronchoplasty in the acutely ill, chronically ill, moribund and/or ventilator-dependent patient. In this manuscript, we present our experience with a series of 12 patients with both short- and long-term outcomes in regards to the performance of tracheobronchoplasty for surgical correction of TBM in patients with severe acute and/or chronic comorbidities, to include morbid obesity, ICU status, ventilator dependence, etc.

## METHODS

### Ethical statement

All patients and/or their surrogate healthcare decision maker included in this series provided informed consent in which they were counselled on the procedure itself, the risks of the procedure(s) and provided consent for intraoperative photographs and/or video recording.

This series is comprised of 12 TBM patients with American Society of Anesthesiologists (ASA) status 3–5. Candidacy was based on bronchoscopic findings during spontaneous ventilation with >90% collapse of the trachea and both mainstem bronchi during expiration (Video 1). We also utilized a dynamic computed tomography scan as an adjunct in patients whom were not mechanically ventilated (Table 1).

**Table 1:** Preoperative, intraoperative and postoperative data of the patients

Patient #, age and BMI	Comorbidity and preoperative ICU stay and ASA	Postoperative ICU stay (days)	Approach	Surgical technique	LOS after surgery (days)	BORG dyspnoea scale at long-term follow-up
1, 48 years, BMI: 55	Trachea stent, bilateral pneumonia, pre-operative ICU + ASA: 5	6	Thoracotomy VV ECMO	PM	7	36 months BORG: 4
2, 61 years, BMI: 62.5	Bilateral pneumonia, CHF, Preop ICU + ASA: 5	13 (complicated by sacral decubitus requiring diverting colostomy)	Thoracotomy VV ECMO	PM	22	36 months, deceased due to CHF
3, 75 years, BMI: 16	Bilateral pneumonia, thyroid cancer, pulmonary and mediastinal metastasis, pre-operative ICU + ASA: 5	5	Thoracotomy	PM	8	21 months, deceased due to metastatic cancer
4, 76 years, BMI: 37	Atrial fibrillation, stroke, CAD, pneumonia, pre-operative ICU – ASA: 4	1	Robotic	PM	4	18 months, BORG: 1
5, 48 years, BMI: 38	Diaphragmatic plication surgery (3 years prior), Pre-operative ICU – ASA: 3	1	Robotic	PM	4	20 months, BORG: 2
6, 68 years, BMI: 36	Covid ARDS, stroke, CAD, CABG, carotid endarterectomy, renal stent pre-operative ICU + ASA: 5	4	Thoracotomy	PM	–	Deceased, POD#3(CMO–family decision, extubated)
7, 62 years, BMI: 38	Respiratory failure, HHR, asthma, PCI, pre-operative ICU + ASA: 4	1	Robotic	PM	4	18 months, BORG: 5 ICU need due to mesh obstruction
8, 67 yo, BMI: 30	CAD, PCI ×5, CHF, CKD, aortic stenosis, pre-operative ICU – ASA: 4	3	Robotic	PM	4	22 months, BORG: 4
9, 41 years, BMI: 32	Down syndrome. CKD, haemodialysis, DM, Previously tracheal stented, preoperative ICU + mechanically ventilated ASA: 4	1	Robotic	PS	7	8 Months BORG 3
10, 77 yo, BMI : 47	CHF, valvular heart disease, PE, O_2_ dependent, COPD, dysphagia, pre-operative ICU – ASA: 3	1	Robotic	PLS	4	1 month BORG 2
11, 62 years, BMI: 46.4	Atrial fibrillation, COPD, CPAP, DM, O_2_ dependent, preoperative ICU – ASA: 3	1	Robotic	PLS	4	2 months, BORG 2
12, 75 years BMI: 29.3	Atrial fibrillation, COPD on 3 l, previous right middle lobectomy 2020, recurrent NSCLC in right upper lobe, completion lobectomy with TBM surgery ASA: 3	1	Robotic	PM	12	8 months BORG 3

ASA: American Society of Anesthesiologists; BMI: body mass index; CAD: coronary artery disease; CHF: congestive heart failure; CKD: chronic kidney disease; COPD: chronic obstructive pulmonary disease; CPAP: continuous positive airway pressure; DM: diabetes mellitus; HHR: hiatal hernia repair; ICU: intensive care unit; NSCLC: non-small cell lung carcinoma; PCI: percutaneous coronary intervention; PLS: pledgeted sutures; PM: posterior mesh; PS: posterior sutures only; TBM: tracheobronchomalacia.

Four patients had a body mass index over 40 kg/m^2^ (Table 1). Four patients had an ASA score of 3 preoperatively, 4 patients had an ASA score of 4 and 4 patients had an ASA score of 5.

Indications for the surgeries for the ASA 3–5 comorbid patients included improved quality of life for patients with tracheobronchomalacia (TBM) refractory to conservative measures; to improve overall respiratory status and in patients with TBM who were felt to have failure to wean and liberate from the ventilator secondary to TBM and other comorbidities. The surgical technique performed in each particular case was not standardized and was based on factors to include patient condition, the general overall health of the patient, ICU status, ventilator settings and the ability of the patient to tolerate single lung ventilation.

In the ASA 3 patients, the indication tended to be more to improve quality of life related to their TBM process; i.e. to improve respiratory status, orthopnoea, to potentially reduce hospital admissions related to respiratory compromise secondary to TBM as each of these patients tended to have multiple hospital admissions related to respiratory issues caused by TBM. In regards to the ASA 5 patients, the surgeries were performed due to the lack of all less invasive methods to improve respiratory status. These patients were intubated and mechanically ventilated in the ICU. These patients did not improve with less-invasive measures, and these patients were not thought to be extubated without the TBP surgery. All 3 of the ASA 5 patients included in our study were in the intensive care and failed to progress in their clinical course secondary in large part to their TBM.

Our desired operative approach was a complete portal robotic approach for those whose operation was performed on an elective basis. A right thoracotomy approach was utilized for patients who were hospitalized and mechanically ventilated, as these patients required high oxygen settings and high airway pressure settings. Extracorporeal support was used intraoperatively in 2 patients who were unable to tolerate lateral decubitus position with single lung ventilation. Patients operated via a thoracotomy approach without extracorporeal support are cases where the patient was not able to tolerate single lung ventilation. Thus, these patients underwent surgery with bilateral lung ventilation, and surgery was performed with appropriate lung retractions.

The cases regarding the 2 patients (patients 1 and 2) in which venovenous (VV) extracorporeal membrane oxygenation (ECMO) support was used: the decision to utilize VV ECMO support in these cases was made preoperatively. VV ECMO was planned for in these cases secondary to the preoperative mechanical ventilation requirements in these patients in which high ventilatory pressures were required for adequate oxygenation and ventilation. These patients also had bilateral pneumonia. Considering these patients could not tolerate lateral decubitus positioning or single lung ventilation, the use of VV ECMO was planned for in the preoperative setting. ECMO is available at our facility at all times.

## RESULTS

All patients within our series had severe metabolic, cardiac and pulmonary comorbidities. Four TBP’s were performed via a right thoracotomy with bilateral lung ventilation and 8 were repaired via the completely portal robotic approach (Table 1). Two patients required ECMO support. Nine patients received TBP of the membranous trachea and both mainstem bronchi with prolene mesh support. The remaining 3 patients had either purse string sutures or suture repair via support with felt pledgets. The membranous support with mesh approach was revised after migration of one mesh into the trachea. In the attached video, the TBM surgery without the use of prolene mesh membranous support is demonstrated (Video 2).

In our series, all patients were liberated from positive pressure ventilation with marked improvement in symptoms. All of the patients who underwent a completely portal robotic approach were discharged without complications. The 2 patients who required extracorporeal support during surgery were both separated from ECMO on postoperative day 2 and subsequently discharged from the ICU. Three patients had died at follow-up.

**Figure 1:**
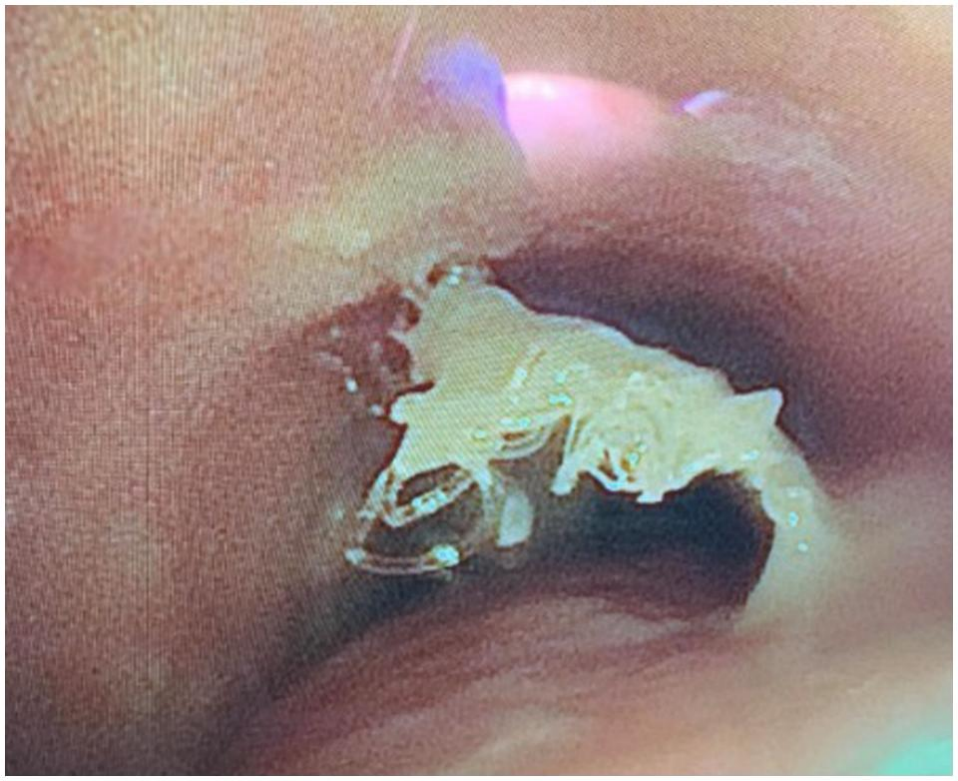
Intratracheal migration of mesh was endoscopically removed.

In 1 patient, the prolene mesh migrated into the trachea causing obstruction, and was removed endobronchially ∼2 years after the operation (Fig. 1). This patient remains with a tracheostomy, the only patient in the series to remain with tracheostomy support.

The mean follow-up for the ASA 3 patients was 7.75 months. All ASA 3 patients reported an improved quality of life in regards to their breathing and respiratory status with an average BORG dyspnoea score of 2.25. The mean follow-up for the ASA 4 patients was 16.5 months. The average BORG dyspnoea score for the ASA 4 patients at follow-up was 3.25. One patient in the ASA 4 category had erosion of the prolene mesh into the airway and required multiple endobronchial debridement procedures, tracheostomy and tracheal stenting. The mean follow-up for the ASA 5 patients was 23.25 months. Thus, the ASA 5 patients demonstrated the best outcomes.

The average length of stay following the surgery for all patients was 7.27 days; with the average length stay following surgery for the ASA 5 patients being 12.33 days. One patient (ASA score of 5) died on postoperative day 3 due to family members’ choice of comfort measures only. The percentage of cases performed robotically was 66.7% (8 of 12 cases) (Table 2).

**Table 2:** Median age, BMI, percentage of cases performed robotically, ASA classification and percentage of morbidity and mortality.

Median age	Median BMI	% Robotic	ASA 3	ASA 4	ASA 5	% Morbidity	% Mortality
64.5 years	37.5	66.7%	4	4	4	25%	30-day: 8.3% Overall: 25%

ASA: American Society of Anesthesiologists; BMI: body mass index.

The percentage of morbidity was 16.6% (2 of 12 patients) in our series. The percentage of 30-day mortality was 8.3% (1 of 12 patients); while the percentage of mortality during the follow-up was 16.6% (2 of 12 patients) due to reasons other than TBM (Table 3).

**Table 3:** Morbidity and mortality outcomes, mean follow-up time

Morbidity %	Mortality %	Mean follow-up time (months)
25% (3 of 12 patients)	30-day: 8.3% (1 of 12 patients) Overall: 25% (3 of 12 patients)	15.8

## DISCUSSION

Severe TBM is gaining recognition as an important entity in ICUs. TBM diagnosis in an intubated patient necessitates bronchoscopic evaluation while the patient is spontaneously breathing and dynamic computed tomography scan to constitute a thorough work-up for evaluation of potential benefit from TBP. Currently, surgical options are almost never discussed in acute care settings, such as in the cases of intubated and mechanically ventilated patients. We believe our strategy described in this series may potentially offer definitive management in patients with severe TBM who also carry severe comorbidities to include morbid obesity, and in those patients who remain difficult to wean from mechanical ventilation.

In addition, recent advances in TBM surgery, such as robot-assisted techniques [3] and posterior mesh tracheoplasty [4], have added to the armamentarium of minimally invasive surgical options that may improve outcomes in this patient population as well.

In patients who have concomitant pneumonia and respiratory failure, single lung ventilation is often not possible. This is particularly true for a right-sided approach that is reliant on single left lung ventilation. In our series, we demonstrate the utilization of VV ECMO in 2 cases in order to safely stop ventilation and allow for a clear operative field.

Although 4 of 12 patients were morbidly obese; these morbidly obese patients endorsed and improved quality of life in regards to their respiratory status following the operation. Two of the 4 patients with body mass index >40 were ventilator-dependent at the time of surgery.

In our series, there was 1 case (patient 3) who could not tolerate single lung ventilation and had tracheostomy. This patient was not obese, as the patient’s body mass index was 16. The surgery was performed via a right thoracotomy with bilateral lung ventilation, with manipulation of the ventilated right lung to expose the trachea and bilateral proximal mainstem bronchi to perform tracheobronchoplasty with posterior mesh placement.

We performed prolene mesh placement in the robotic-assisted cases; however, 1 patient in our series (patient 7) developed mesh erosion into the tracheal lumen. This patient required multiple endobronchial debridement and extractions of the mesh, as well as tracheal stent placement and tracheostomy secondary to this complication. Following this case, we tended to perform both the pledgeted suture tracheoplasty (Video 3) and posterior sutures only tracheoplasty in patients in whom a short TBM surgery is required, such as in the case of the patient who required concomitant lobectomy. Bronchoscopy after pledgeted suture demonstrated significant outcomes (Video 4).

Prior to performing TBP with posterior mesh on patient 6, who had an ASA 5 score, an extensive discussion was had with the family to include the possibility of long-term need for mechanical ventilation following the operation. The medical decision maker(s)/family did elect to have the surgery performed for this patient. However, on POD#3, they changed their decision and wished for the patient to be made comfort measures only. Thus, he was extubated and expired on POD#3.

We feel our series demonstrates that the surgical treatment of TBM in significantly comorbid (ASA 3–5), mechanically ventilated, super morbidly obese patients may permit weaning off ventilator support. In this regard, there are 3 salient points worth underscoring when evaluating the patient for TBP:

1. 90% collapse on bronchoscopy under spontaneous ventilation is diagnostic of TBM in an intubated patient.
2. ECMO prior to surgery allows for single lung ventilation and greater intraoperative stability.
3. ECMO and tracheotomy decannulation may be performed once patient is stable.

Treatment of TBM in super morbidly obese patients could be considered an indication for tracheobronchopexy/TBP in patients that are unable to be weaned from mechanical ventilation.

In conclusion, the repair of TBM in patients with multiple comorbidities and with severe life-threatening problems within or outside the ICU setting may lead to improvement in the ability to wean from positive pressure ventilation. Surgical technique and use of mesh support in TBP may differ in an attempt to decrease the duration of the surgery in patients with severe comorbidities. Thus, TBP may be a promising surgery to consider in patients with multiple comorbidities. However, contribution to the long-term survival still needs to be evaluated.

**Conflict of interest:** none declared.

## Data Availability

The data underlying this article will be shared on reasonable request to the corresponding author.

## Notes

Presented at the EACTS Annual Meeting, Vienna, Austria. The abstract for this manuscript won best E-poster in the general Thoracic Field at the 2023, EACTS Annual Meeting.

## ABBREVIATIONS

ASA: American Society of Anesthesiologists status
ECMO: Extracorporeal membrane oxygenation
ICU: Intensive care unit
TBM: Tracheobronchomalacia
TBP: Tracheobronchoplasty
VV: Venovenous
